# A Qualitative Investigation of Clients, Significant Others, and Clinicians’ Experiences of Using Wireless Microphone Systems to Manage Hearing Impairment

**DOI:** 10.3390/audiolres12060059

**Published:** 2022-10-27

**Authors:** Nerina Scarinci, Mansoureh Nickbakht, Barbra H. Timmer, Katie Ekberg, Bonnie Cheng, Louise Hickson

**Affiliations:** 1 School of Health & Rehabilitation Sciences, The University of Queensland, St Lucia 4072, Australia; 2 Sonova, 8712 Stäfa, Switzerland

**Keywords:** wireless microphone systems, decision making, rehabilitation, adult hearing impairment, significant others

## Abstract

This study aimed to explore the perceptions and experiences of adults with hearing impairment (HI), their significant others (SOs), and clinicians regarding the use and provision of wireless microphone systems (WMS). A qualitative descriptive methodology was used, with a total of 43 participants across three groups: (1) 23 adults with HI who used WMS; (2) 7 SOs of adults who used WMS; and (3) 13 clinicians who provided WMSs to adults with HI. Participants completed an individual semi-structured in-depth interview to explore their experiences, with the data analysed using thematic analysis. The analysis revealed five themes encompassing the perceptions and experiences of WMSs: (1) with experience and clear expectations, users believe that WMS can make a difference; (2) the trial and decision-making process is important; (3) clients’ experiences using WMS; (4) issues with WMS and technology; and (5) users require ongoing training and support to use WMS. These findings highlight the complexities of providing and using WMS with adults with HI. However, clients, SOs, and clinicians all reported that, with appropriate experience, expectations, training, and support, WMS can make a real difference in listening and communicating in different situations. There is also an opportunity to involve SOs more throughout the rehabilitation process.

## 1. Introduction

Hearing aids—or, sometimes, cochlear implants—are typically recommended as the primary treatment option for adults with hearing impairment (HI). However, some adults, particularly those with severe and profound HI, still report difficulties with hearing in noisy environments or when a speaker is at a distance [1,2]. These adults can benefit from using assistive listening devices (ALDs) in addition to their hearing aids and/or cochlear implants. Among the many types of ALDs available, wireless microphone systems (WMS) are the most common [3,4]. These WMS include ALDs such as remote microphones and FM systems; however, for the purposes of this paper, the term WMS will be used to include the range of ALDs available. Adults with HI report using WMS in a variety of listening situations, including conversations in noise (e.g., group conversations), phone conversations, in cars, and listening at meetings or in church [5].

Most research into the effectiveness of WMS has focused on measuring benefits in clinical and laboratory settings. Improved speech recognition has been found in some studies of adults with mild and moderate HI when hearing aids + WMS were compared to unaided and hearing-aid-alone conditions (see the systematic review by Maidment et al., 2018) and in studies of adults with greater degrees of HI when hearing aids and/or cochlear implants + WMS were compared to amplification alone [6,7,8,9,10,11,12,13,14]. Most recently, such improvements in speech recognition have also been reported for adults with single-sided HI using cochlear implants [15,16]. There is also evidence of self-reported benefits derived from using WMS from studies using self-report questionnaires and written journal entries [5,10,17]. This research informed the development of a clinical tool for assessing adult cochlear implant users’ perceived benefits of using WMS during a trial period [17], where increased satisfaction in certain listening situations was reported by some but not all users [5,17]. Another study compared self-rated quality of life for hearing aids + WMS to hearing aids alone and found no significant differences [5].

Interestingly, despite the generally positive research findings to date, adults with HI typically did not report wanting to purchase WMS at the conclusion of a trial period [5,17,18]. The reasons cited for this included disappointment with the lack of benefits in group situations, the stigma associated with the overt appearance of WMS, inadequate education and training on how to use WMS, and cost. Studies to date have used self-report questionnaires and written journal entries to understand the barriers and facilitators to the use of WMS, and little research has explored user perceptions and experiences in more detail. Therefore, the aim of this study was to conduct a qualitative interview study to investigate the perceptions and experiences of adult clients with HI, SOs, and clinicians regarding the use and provision of WMS to adults with HI. The advantage of an interview study in this context was that richer and more accurate data were able to be obtained, as it allowed the interviewer to respond in real-time to participant reports by asking further questions that probe specific, relevant topics [19].

## 2. Materials and Methods

### 2.1. Design

A qualitative descriptive methodology was used in this study. Individual in-depth, semi-structured interviews were conducted with adult clients with HI (“clients”), the SOs of adults with HI (i.e., spouse/partner, family member, close friends), and hearing care professionals (“clinicians”) who used or recommended WMS to adults with HI. A qualitative approach was selected as the most appropriate methodology, as it is an effective means of capturing and thoroughly exploring the perceptions and experiences of healthcare users and healthcare professionals [20].

### 2.2. Participants

Participants were recruited from Australia and New Zealand through email distribution, social media advertising, and word-of-mouth via professional and informal networks (including hearing aid manufacturers, professional associations, and government and private audiology clinics and programs). Participants with HI were eligible to participate if they had been diagnosed with a severe to profound HI. We excluded participants who had an evident cognitive impairment, were highly dependent on carers, were under the age of 18 years, lived in an aged care facility or other supported care accommodation, did not speak English as their primary language, or were otherwise unable to participate fully in an individual interview.

There were a total of 43 participants across three participant groups: (1) 23 adult clients with severe to profound HI (see Table 1); (2) 7 SOs who were the key communication partners of an adult with HI who had experience with WMS (4 partners, 2 mothers, and 1 housemate); and (3) 13 clinicians who provided WMS to adults with HI. Clients ranged in age from 21 to 80 years (mean = 55.62; SD = 17.69) and consisted of 13 females and 10 males. Eight of the twenty-three clients with HI had congenital pre-lingual hearing impairment. One of the clients (Client 19) was also an audiologist with four years’ experience with using WMS; however, for the purpose of this study, this individual was classified as a “client”, as they spoke in the interview from the perspective of a client who used a WMS, rather than a clinician. Six of the seven SOs were female, and the group ranged in age from 52 to 66 years (mean = 58.57; SD = 5.53). Six of the seven SOs were connected to the adult clients interviewed in this study. The adult client related to the seventh SO was excluded from the study, as the client had complex communication needs and was not able to participate in an individual interview. The clinicians comprised eight females and five males with a range of years of practice experience: four clinicians had 2–5 years’ experience, three had 6–10 years, four had 11–20 years, and two had more than 20 years’ experience. One of the clinicians (Clinician 9) interviewed in this study also had an HI and used a handheld/body-worn WMS; however, for the purposes of this study, this individual was classified as a “clinician”, as they did not use their WMS regularly and spoke primarily from the perspective of a clinician who provided WMS to clients.

### 2.3. Study Procedures

Individual semi-structured interviews occurred in-person with participants at their home, at their usual audiology clinic, at another mutually agreed-upon location, or via teleconference. All interviews were conducted by the fifth author, a researcher who was unknown to the participants. The interviews followed a topic guide, with clients and SOs asked to reflect on their experience, their reasons for using WMS, the benefits and challenges, and the training and support needs (see Appendix A). The clinicians were asked about their knowledge and experience in recommending and fitting WMS, the perceptions of benefit, barriers, and facilitators to recommending systems, barriers and facilitators to client use, and training and support needs. Follow-up questions were asked where further clarification of the main topics or the participants’ responses was required or to elicit a more comprehensive response from the participant.

Interviews ranged in duration from 19 to 91 min for clients, 17 to 33 min for SOs, and 10 to 53 min for clinicians. At the end of the interview, the participants received a gift card (AUD 25) as a token of appreciation for their participation in the study. The interviews were audio-recorded and transcribed verbatim by a professional transcription service, with the interviewer checking the audio recordings against the transcripts for accuracy. The recruitment of participants stopped once we reached a saturation of data, wherein no new codes or themes emerged from the data [21].

### 2.4. Data Analysis

Reflexive thematic analysis was used to analyse the interviews. Thematic analysis is a method of identifying, analysing, and reporting themes within the qualitative data and is an established research method used frequently in health research [22]. Six steps of thematic analysis were followed: familiarisation with the data, generating preliminary codes, searching for themes and subthemes, reviewing themes, labelling and defining themes, and creating the report [22]. First, the second author read the transcripts to familiarise herself with the data and de-identified them. The data were managed using NVivo software [23], which is designed to support the organisation and analysis of qualitative data. This researcher generated the initial codes related to the research aim, sorting these initial codes into categories and subcategories. The first and second authors then examined the relationships among the data items and sorted the initial codes into categories, subcategories, and themes, which were reviewed by the entire research team. The rigour and the trustworthiness of the analysis were addressed in regular meetings for peer debriefing and double-checking of the coding. The authors discussed the coding scheme and overarching thematic analysis until the team reached a consensus. The quotes reported in this paper represent a few examples of each category and subcategory.

## 3. Roger miniMic—Clip-on Wireless Microphone

Five themes were identified from the interview data, namely: (1) with experience and clear expectations, users believe WMS can make a difference; (2) the trial and decision-making process is important; (3) clients’ experiences with using wireless microphone systems; (4) issues with WMS and technology; and (5) users require ongoing training and support to use WMS. These themes, categories, and subcategories are summarised in Table 2, Table 3, Table 4, Table 5 and Table 6, with additional participant quotes presented in the Supplemental Digital Content (SDC). The clients and SOs typically described their WMS by the brand name (e.g., Roger^TM^); however, these brand names have been anonymised, and a broader description is used in the analysis presented below (e.g., handheld/body-worn WMS). The clinicians tended to use the term “FM systems” to include all WMS.

**Theme** **1.** *With experience and clear expectations, clients, SOs, and clinicians believe that WMS can make a difference.*

All users expressed a general belief that WMS had a range of benefits, with some clients and SOs indicating that using the devices made a significant difference in their lives: “*I got the [wireless] receivers, and the [Handheld-body-worn WMS] initially, and it was just a game-changer for me*” (Client 23).

Users reported that the WMS were multifunctional, with “*wide application*” (Client 11) for meetings, study, background noise, phone, TV, computers, healthcare services, home, social activities, sport, transport, and when lip-reading was not possible. The main use of WMS was reportedly for meetings, with one client describing how she “*couldn’t hear them [attendees] from there to there*” before getting a WMS, but now she felt “*a lot more confident being able to handle the meetings*” (Client 1). Clinicians also described WMS as “*great for clients for meeting goals where they want to hear over distance*” (Clinician 2). Some clients reported that they only used WMS at work, as they “*can’t see a big use for it outside of work*” (Client 1).

Educational settings presented another beneficial listening situation for WMS, including lectures, university classes, professional development activities, bible study, and conferences. For example, Client 2 said: “*It’s been great, I could not have done schooling or university or all the rest without it*”. Clinician 4 also described the system as beneficial for the educational context because “*[clients] might go to a lot of classes where they can’t always sit close to the instructor, like a pottery class, line-dancing, yoga*”.

Another key listening situation was hearing in background noise. Although some clients reported that “*The [WMS] doesn’t cut out all the background noise … it does reduce it*” (Client 22), the adult with HI who was also a clinician noted that she used WMS successfully in background noise.

*I’m in … a really really noisy environment … not an ideal situation for somebody with a hearing loss, but through what I’m getting from the [WMS], that actually means that I can work quite confidently in that sort of noisy environment*.

(Client 19)

Using WMS for the phone was also reported to be very helpful: “*I work as a lawyer, and I’ve always struggled with being able to use the telephone at work … If [WMS] is on and working I don’t miss calls, because it beeps to let me know when calls are coming through. I can actually have conversations with people easily over the telephone*” (Client 23).

Clinicians reported varied experience with WMS, from limited to extensive experience with WMS from different manufacturers: “*I think I fitted probably over 150 wireless devices to adults with CIs [cochlear implants] … I haven’t done enough of them [WMS], to be honest. We’ve only done a couple of [WMS]*” (Clinician 4).

Being fitted by an experienced clinician was as an important consideration for clients: “*Their [HA/WMS manufacturer] lovely representative … she was doing the fitting, and I tell you what, a very skilled audiologist doing your fitting from the company makes a huge difference to the result*” (Client 21).

Another client said:

*It was actually the first one that my audiologist had anything to do with, and she didn’t connect it [WMS] properly. And so for a while, I was really frustrated because I thought it was me not understanding the technology … So when I eventually went back to her, she worked it out and it was all right*.

(Client 24)

Clinicians had different beliefs about clients who were more likely to be successful users of WMS. Specifically, some clinicians believed that “*age and cognition are a big factor*” (Clinician 13), and, thus, younger and “*technologically savvy*” clients and people without dexterity and vision impairment would be more successful users. However, a clinician also noted the importance of an SO’s support in helping older clients use wireless devices successfully: “*If they have to press more than one button … then it’s less likely to take off, unless they’ve got their kids around a lot and are able to do it for them*” (Clinician 11).

User expectations were another important factor influencing the success of WMS, with some users reporting that the devices met all their expectations.

*I think it’s gone beyond my expectations because I’ve had … quite a few FM systems growing up, throughout kindergarten, primary school, and high school. And I think the functionality and the ease-of-use has been a lot more efficient and beneficial to my life than previous FM systems have been*.

(Client 16)

However, some users noted that they had higher expectations that were not met by their WMS: “*even though I’d had it for two years, it’s still not my normal*” (Client 18).

Community understanding of WMS and how to communicate with them effectively was identified by participants as a factor influencing their success: “*The FMs, the [WMS] or loop systems, they have all met my expectation … but 99 times out of 100, it’s the way other people use the equipment that renders it not as beneficial as I know it can be*” (Client 12).

Greater public awareness was desired, and clients believed that “*if you don’t speak up, people don’t learn*” (Client 12); therefore, a client provided a suggestion for manufacturers:

*If [manufacturer] wants to do something in the broader education, it’s probably not a bad thing … A device and FM might be mine, but I’m only one part of a conversation … maybe more needs to be done from the other way*.

(Client 2)

However, a clinician who did not perceive public awareness as a major issue said:

*That awareness side of things, probably I would say, that only comes up 15–20% of the time. It’s not a massive issue for a lot of people, but it is for some*.

(Clinician 2)

**Theme** **2.** *The trial and decision-making process is important.*

The trial and decision-making process was reported to play a crucial role in the introduction of WMS to their uptake and use (see Table 3). Clinicians reported that they identified potential users for trialling WMS based on client needs and goals.

*I try to find the main need and be specific and try to figure out what they really need it [WMS] for. Like if it’s just the TV that their struggling with, something like a TV streamer would be better than having all of these extra devices on top. But some people do want all the extra bells and whistles, and I think as long as they can use it [WMS] and handle it, that’s fine as well*.

(Clinician 3)

However, almost one-third of the clinicians who participated in this study reported having certain views about which clients should be introduced to WMS. For example, clinicians introduced WMS “*for anybody who has higher listening needs*” (Clinician 6) or if they perceived that hearing aids were not enough:

*If they had also trialled hearing aids previously and hadn’t had a lot of success, particularly in groups or noisy situations, that’s probably where I’ve looked at introducing it [WMS]*.

(Clinician 5)

Clients also described how they had been introduced to WMS, mostly in audiology clinics: “*I was primarily complaining about the fact that I couldn’t enjoy music, so he [audiologist] suggested I use the [WMS], so I did, and bingo it was good*” (Client 11).

Some clients explained how they learned about WMS from sources other than clinicians, including marketing materials, friends, deaf organisations, and employment agencies for people with disabilities.

*They’re not very common down here, and I had never heard of them [WMS], so a friend said: “oh, you should get one of those [WMS].” I said: “what is it?” … I was working with her, and she has a daughter who has one [WMS], and she said: “Oh yeah, that’s a good thing*”.

(Client 20)

A couple of clients also talked about the impact of online reviews in helping them make decisions: “*There was just very good reviewing on the [WMS], just listening to people’s feedback online. It just made me think it was certainly the way to go*” (Client 14).

Some clients and SOs reported that they made the decision to obtain a WMS because they experienced pervasive hearing difficulties (e.g., difficulties with lip-reading, background noise, shopping, transport, watching TV, and answering phone calls).

The trial period reportedly allowed for an opportunity to demonstrate the benefits of WMS for clients and SOs and help inform users’ expectations: “*They let me do a trial and that was very very helpful indeed*” (Client 21).

A successful trial also then supported clients and SOs to make a decision to obtain a WMS: “*You know, the trial will sell the device, especially if you talk with the patients about the specific places where they’re likely to get benefits*” (Clinician 4).

However, the interview data suggested that there was no unique instruction to guide the trial and fitting process. One clinician checked the fitting by going out of the room and talking to the client: “*She went out of the room about six times and spoke to me from outside the room. Just to make sure that yes, everything was going good*” (Client 8).

Another clinician practiced with clients and then asked them to go to a coffee shop to try the device. She explained: “*normally in the fittings, sometimes we’ll let people go off with the device if they’ve got someone with them to see how it goes*” (Clinician 3). This clinician also reported that she asked clients to bring their SO to the fitting session to make sure the client could hear them when using the WMS.

Whilst a long trial period was desired by clients, clinicians reported varied trial durations, ranging from one day to one month. A client who was offered a short trial said: “*We’d need to have far more trials*” (Client 2). Clinician 1 had a suggestion for clients to make the most of short trials, noting:

*I’d always ask them to identify a period when they’re going to be more likely to use it and situations where it’s going to benefit them. So going back to their COSI’s [Client Oriented Scale of Improvement], right you’ve got a meeting once a month … So, let’s book a trial around that period where you’ve got your meeting, so you get in the day before we figure out how it works, show you how to charge it, so you go out you experience it, you come back a few days later.*

In addition to trials, cosmetic considerations also influenced clients’ decision making. Most users reported that they liked the pen shape, light weight, and small size of one particular WMS, the Roger^TM^ Pen, and reported that: “*they’ve done a great job in helping it [Roger^TM^ Pen] blend in with the environment, rather than something that sticks out and people are asking about it*” (Client 16). A client also expressed her belief that the pen shape of the Roger^TM^ is “*less intrusive*” and may prevent clients from experiencing prejudices in society: “*A really important thing, is that the equipment that people are using needs to be inconspicuous or in such a way that people don’t notice it as being anything different from what someone else is using like a pen, for example*” (Client 10).

The compatibility of a WMS with other hearing devices was also perceived as an important factor in decision making. A client said: “*because I’ve got a [other manufacturer] hearing aid and cochlear implant, they said the Roger^TM^ Pen would be the best because it feeds to both*” (Client 8). Clinicians also reported that “*in terms of actually pairing it [WMS] up with your implants, it’s extremely easy*” (Clinician 9). Another client also reported that the compatibility of her WMS with another manufacturer’s equipment makes her “*life so much easier, and it helps keep the cost down for everyone*” (Client 4).

SOs also reportedly made an important contribution to the decision-making process for purchasing a WMS. Clinicians reported their desire to involve SOs in appointments given that “*two heads are better than one*” (Clinician 2). Clinicians noted that involving SOs is “*a really good way of managing expectations not only of the device and the hearing loss but also on the person … If the SO perceives that just putting on a listening device is going to solve all their problems, they’re less likely in the long term to be supportive of that person*” (Clinician 1). Clinicians also reported that involving SOs is important to the ongoing management of the device, “*[s]o that they [SOs] know how to manage it if the client struggles at any point or becomes incapacitated*” (Clinician 5).

However, only some SOs said that they were involved in appointments, with others reporting that they had not been involved in trials or decision making and had not received enough information. One SO said:

*I don’t think I’ve ever received any support or information about it [WMS]. He’s been the one who’s educated me and he’s been the one who’s educated others, but I specifically haven’t been told how to use it, or what it’s for, or why people use it*.

(SO 4)

The relatively high cost of devices was reported as a barrier to uptake, with a client noting: “*the biggest obstacle is money*” and “*I would have got it far sooner if it hadn’t been for the price*” (Client 22), but a clinician believed that, given the multi-functionality of the particular WMS, “*you get what you pay for*” (Clinician 11). Adult children of clients may have varied points of view about cost. As Clinician 10 described:

*Some of them [adult children] are worried about the money and “what are you spending my inheritance on?” And some of them are like, “well, you know, Mum, it’s your hearing. You gotta be able to hear. If we need to spend a bit more money so that you can hear well when we all come and visit and whatever else, then that’s what you got the money for”.*

Some users benefited from government funding, and this was reported as a motivator for obtaining WMS. As a clinician said: “*I guess through DVA [Department of Veteran’s Affairs], patients are also a little bit more motivated because it’s of no cost to them … It’s great for me and great for them and everyone’s sort of happy*” (Clinician 3).

**Theme** **3.** *Clients’ experiences using wireless microphone systems.*

Users reported different experiences when using WMS, ranging from improved sound quality and clarity and less listening fatigue to improved confidence and quality of life (see Table 4). Users also reported feeling more confident and socially included when using the WMS: “*I think people tend to feel a lot more included when they use it because they’re able to use that device in a way that gives them more control*” (Clinician 2). SOs also reported improvements to their quality of life, noting that the WMS was “*a tremendous help*” in “*personal communication*” (SO 1), reducing their frustration, as they no longer needed to raise their voice when communicating with the client.

Users reported that they learned about using WMS in different situations by “trial and error”: “*It was kind of once she’d [audiologist] shown me what to do and it was me just sort of doing the trial and error, how far apart to put the table mics and whether I worried about that one or not* (Client 1). Clients reported that WMS allowed them to partake in additional tasks such as note-taking in lectures: “*It [WMS] basically enabled me to have my head down and working instead of always looking up and looking at the person speaking and trying to lip read them because if I was doing that I couldn’t physically write*” (Client 4).

Clients discussed the range of people’s reactions to WMS: “*people make different comments when you ask them to put it [microphone] on*” (Client 5). Some clients reported that other people were supportive, accepting, and positive of WMS: “*The [meeting] chair, I give the WMS, and he wears it around his neck, very happy to do that*” (Client 1). A few clients reported curiosity among some people (e.g., engineers and musicians) about WMS.

*I went to a restaurant with very old friends in [city] and I just popped it [WMS] down on the table hoping to catch up with them after quite a long time. And he’s a musician and so he’s been around sound systems and all the rest of it. And he just wanted to stop the conversation until I’d explained exactly how it works …*

(Client 21)

Some participants reported that other people were unsupportive of the WMS, explaining that others had not been willing to use the WMS:

*Some people are reluctant to use it [body-worn WMS] … they don’t want to wear it around their neck … People have complained that they don’t like wearing it … They don’t understand the need for him.*.

(SO 4)

Participants also reported that others had legal concerns about being recorded after assuming that the WMS was an audio-recorder: “*People seem to be uncomfortable sometimes with that request. Maybe they feel they’re being recorded. Maybe they felt it’s an intrusion*” (Client 12).

Some clients, therefore, used specific approaches to communicate with others about using WMS (e.g., explanation, using humour, or not offering information). A couple of clients, for example, reported that they explained to others how WMS work: “*at the time, she refused to wear the FM until it was explained clearly that it doesn’t amplify any for anyone else it was for mine and mine only at which point then she had no problem*” (Client 2).

Finally, an analysis of the interview data revealed that almost one-third of the clients were using their wireless devices regularly; however, the remaining clients did not use their devices consistently or only in specific situations: “*very much, it will depend on the situation, the people I know, and the flow of conversation*” (Client 21). Others reported that they did not use their WMS anymore: “*I hardly use it [Clip-on WMS] now because I don’t find it very effective*” (SO 7). A client, however, expressed her belief that: “*It’s like anything, you’ve got to use it to get used to it, so the more you use it the better at using it you get. So that’s the point I’m at. I’ve got to use it more to really be happy with just picking it up and using it*” (Client 13).

**Theme** **4.** *Issues with WMS and Technology.*

Although most users reported several benefits from using WMS in a variety of situations, some reported issues with WMS and technology that caused frustration (see Table 5). The first main issue was reported to be in group situations. For example, “*[Body-worn WMS] works obviously the best when one person is wearing it*” (Client 5), so “*the more people that are potentially talking, the less benefit it does give you*” (Client 19). Another client reported: “*if everyone was carrying a [body-worn WMS] that I was tuned in to and they spoke one at a time, that would be good but otherwise the practicality just sort of make it a bit unrealistic*” (Client 18).

Frustrations also occurred in meetings when the WMS stopped working: “*With the Pen, it’s like bloody hell, it’s not working, and there’s not really anything you can do about it on the spot. You’re in a meeting, you can hardly say: “Oh look, I’m just going to Google all the information and try and figure out what’s wrong with my device.” You have to just struggle on with what you’ve got at the time*” (Client 3).

Another meeting challenge described by participants was the disruption to the flow of the meetings: “*I guess the flip side about it [handheld/body worn WMS] obviously is that people have to wear microphones and take microphones off and put microphones on … and pass them around. There’s a lot more destructions in the flow of the meetings*” (Client 15).

However, another client had a positive point of view about passing the handheld WMS around in the meetings, as it gave them time to think about what was said:

*If you’re using these devices, a big advantage is … ideally, they have to talk one at a time because they’re passing the microphone, and the idea is that only the person with the microphone talks … it creates that space, that moment that I can spend thinking back over what was said and clarifying it in my own mind*.

(Client 12)

Other communication challenges reported by the clients included hearing reverberations, static noise, and background noise rather than conversations: “*I just don’t think that it [WMS] can filter the noise that you want to hear from the rest of the noise*” (Client 3). Some clients also reported that they experienced robotic and unnatural sound quality with WMS: “*I did not like the sound of my voice when I was speaking to people. It sounded quite robotic, which I didn’t like*” (Client 14). Clients and SOs also reported that WMS sometimes block out the surrounding voices and lock to the speaker or TV. Although one client reported that his clinician was able to adjust the settings so “*he can hear his SOs while watching TV*” (Client 8), another reported that “*he [Audiologist] just can’t see any other adjustments he can make on it*” (Client 7).

The complexity of WMS was another issue for users, with most clinicians noting that the design of the WMS did not necessarily take into consideration the characteristics of the main user groups, namely, older adults:

*I think the people that are designing the products are people like you [interviewer] and me, younger, good dexterity, good vision, you know, healthy. I don’t think that they’ve always considered the populations [older adults] who are gonna to use the devices as well. They’re beautiful to look at and discreet in some instances but manageability is definitely something that I have noticed with the clients, the client populations that I was fitting, they really struggled*.

(Clinician 1)

The complexity in using the device was related to various design and technical issues, including the size and location of the buttons on the WMS, the light-emitting diode (LED) lights, the battery capacity, and issues with connectivity and integration with other devices. The size and location of the buttons was particularly an issue for older adults managing the device: “*It’s dealing with the population who are usually quite elderly. They take a lot of practice to keep that on board*” (Clinician 12), and “*One of the other issues was having the pairing button too close to some of the other controls as well, so it’s very easy to accidentally switch that and activate that as well*” (Clinician 5).

The LED lights on the WMS were reported as challenging because (1) similar colours (e.g., red and orange, blue, and purple) were used instead of “*a wider spectrum of colours*” (Client 16), (2) LEDs were not in line with the switch, (3) LED lights were not obvious in dark situations, and (4) clients had to look up the manual to interpret the meaning of LED colours.

Some users reported challenges with the battery, as “*the battery doesn’t last. And they actually say in the manual it’s not intended for extended use*” (Client 22). Clients also reported that it was difficult not knowing how much remaining charge the WMS had: “*If there was a little gauge or something on the [WMS] that gave me an indication of how much battery life I had before I needed to recharge it again. Something like that would be useful*” (Client 10).

Some users also reported connectivity issues with wireless devices. One of the clients who has experience with a range of WMS said:

*Well, I don’t love the [WMS] to be quite honest with you. I think that’s the one I have the most difficulty with. It seems that sometimes it works and sometimes it doesn’t. So last week I was trying to use it in a meeting, and …I just couldn’t get it to connect*”.

(Client 3)

Integrating the WMS with other devices, including mobile phones, was reported to be complicated for some clients: “*It takes too much time. By the time you switch everything on and you put on the pendant and you turn on the implant and the hearing aid, they’ve hung up … It’s a hell of a lot of steps*” (Client 8). However, some clients reported success in using WMS with their mobile phone: “*… to actually be able to make phone calls in the car because that was absolutely something I could not do before*” (Client 25).

Some users reported that setting up WMS with the television was too complex and that they “*had far less success getting results with the TV streamer … cannot get it set up*” (Clinician 4).

Users expressed a desire for a simpler device: “*What would be ideal is … whatever products are out there become truly integrated with technology and just make it much easier to navigate rather than lots of different bits and pieces*” (SO 3). A client with a technical background expressed his desire to contribute by being “*part of research and development … even just as a guinea pig*” (Client 25).

**Theme** **5.** *Clients, SOs, and Clinicians Require Ongoing Training and Support to Use WMS.*

Although some training and support were available, some users expressed a need for additional information, training, and support for using WMS (see Table 6). Some clients were not aware of the different situations in which wireless devices could be used, so they reported a need for more information.

*I’m talking about understanding how I can get the optimum use of my [WMS] in different situations, so there might be more situations that have come to light that a [WMS] is really quite suitable for. But I’m aware that I can use it on lectures, on the sound systems, in the car. I’d be really interested to know of the other situations that are possible*.

(Client 10)

*But again, there is lack of information. Can it be used for two-way communication on the computer? I don’t even know the answer at the moment*.

(Client 22)

It was also reported that clients may not receive enough information in response to their questions. A client, for example, said: “*I never had any experience, I’ve actually asked, they [audiologists] simply said, that [WMS] is not for you*” (Client 9).

SOs also required increased awareness and information about HI and hearing technologies. As SO 5 said: “*The challenge for me is just to understand it [WMS] better too*”, and Clinician 1 said:

*I think that’s the problem … people go “well we [SOs] just bought hearing aids or we just bought that and we just bought this, why can’t they hear me here?” And it’s just that lack of understanding of hearing loss and its impact on people’s quality of life … You know, it’s just that lack of understanding and that’s probably the worst comment you can tell someone with hearing aids, you know, in my opinion, “just put your hearing aids on so you can hear me*”.

(Clinician 1)

Users also reported a need for additional user-friendly instructions, although there were some written instructions and manuals available. The users provided some suggestions for more visual instructions too.

*I think some very short video tutorials about, for example, how you can connect more than one [WMS] device to your receiver, another one on, you know, the whole connecting to a landline telephone, along with a written tip sheet on the website that actually sets out the process. I think that would be really helpful. It’s really good to see, in a video format, what’s what, I guess*.

(Client 23)

The analysis also revealed that clinicians may require more support as well. Clinicians reported that a lot of counseling is required for fitting wireless devices: “*…if it was someone who wanted it for numerous situations, normally they’ll stick with the device and I just have to tweak it or counsel them quite a lot about that*” (Clinician 3). Clinicians reported that they helped clients with setting up TVs and phones, but it was challenging for them.

*Quite often it’s to do with getting it all connected up to a particular phone which is always a challenging thing when, you know, we don’t know the ins and outs of every phone as well because the connection just keeps dropping out, or something like that. Trying to figure out, is it the [WMS] or is it the phone that’s having the problem*.

(Clinician 6)

Clinicians reported that the HA/WMS manufacturer supported them by phone, email, and representatives, and “*they [HA/WMS manufacturer] always send us out, you know, the pack if there’s an update on it, which is always helpful*” (Clinician 13). Clinicians also reported that they learned about WMS devices from colleagues, conferences, online tutorials, and internships, but some clinicians and clients identified a need for more refreshers and training for clinicians regarding fitting WMS devices.

*It would’ve been great if they could’ve come into the clinic when I had a client and help me to set the first one up, and talk me through the issues. Like, one of the reasons we haven’t had so much follow up with the [HA/WMS manufacturer] ones is that [CI manufacturer] talked me through all the potential things that can go wrong with their wireless devices; the tricks and tips which saved a lot of time. Because if I hadn’t been able to share those with the recipients from the outset, we would’ve wasted a huge amount of time, which would’ve completely undone any profit and actually made it unprofitable. So, the training’s actually important so that you get the fitting right from the start*.

(Clinician 4)

A clinician also suggested some free online courses for audiologists: “*So if it was free and if it was quite, yeah, on some kind of portal through, I don’t know how, through the ASA [Audiology Society of Australia], I think that would be really, really, really good for [HA/WMS manufacturer] to do that*” (Clinician 8).

In addition to clinicians, clients also reported that they required ongoing support and follow-ups for setting up wireless devices in meeting rooms and for TVs and phones. A client who experienced a supportive audiologist and then an unsupportive hearing company described the importance of ongoing support to make her life easier.

*I pity, I feel very sorry for anyone who doesn’t have someone like [audiologist] to help them, because I went to a company for a while—[audiologist] went off on leave for a while—and so for two years I went to a big company, and it was just awful … Every time I went in there it was with someone different, and they just weren’t helpful. They just wanted money, you know? Just awful, the receptionist was always rude. I don’t know if she was overworked but just having [audiologist] look after you makes my life so much easier.*

Clients also needed to learn and remember how to use WMS. A client, for example, said: “*Someone that’s not technology-savvy, like me, I really struggled to actually learn how to use it [WMS], how to link it, what the different settings are*” (Client 23). Another client said: “*The Pen’s okay, nothing wrong with the Pen. Just a matter of me thinking now how do I use this? Which buttons do I push?*” (Client 18).

Furthermore, clients required support for troubleshooting, and they suggested that “*[Hearing device company] could lift its game quite a lot in terms of being providing more information about the things that go wrong*” (Client 21). A client whose work is in the HI area expressed her desire for a longer warranty period, and a couple of clients expressed their desire for a direct line to the HA/WMS manufacturer for troubleshooting.

*I think that’s the biggest issue. I work across several companies in my work. The ones that operate best are the ones that you can have connections to-to troubleshoot, and [HA/WMS manufacturer] doesn’t have a direct line to troubleshoot … we’ve been saying to other professionals that the downfall is that there is no direct contact with the manufacturers. There are at least two other sound field technology companies that have direct input, so you can ring them at any time to troubleshoot. [HA/WMS manufacturer], you can’t do that*.

(Client 15)

Clients wanted to access a direct line because audiologists would not know how to troubleshoot some problems.

*I’ll tell you what, you can’t ring up [HA/WMS manufacturer]. They always say you’ve got to go through your audiologist. And look, I like my audiologist but she’s not probably totally cluey about the technology. She’s sort of maybe someone a little bit maybe not as old as me. But, you know, not that much younger that she’s not really of that generation that understands technology as well*.

(Client 24)

However, the majority of clinicians reported that they offer clients additional appointments for troubleshooting: “*if they had an issue, they could contact us and we would try to walk them through whatever problem was coming up for them*” (Clinician 7) and “*saying to them: ‘If you are having trouble, you can contact me. We can talk you through it or you can come back again and see me’*” (Clinician 9).

A couple of clients also expressed their desire for peer support and for connecting with other wireless system users.

*Like if you’re stuck on a rock face feeling like you’re about to fall to your death, who do you want to help you? A geologist or a rock climber? The geologist will tell you about the rocks, the rock climber will show you what to do. The audiologists are like the geologists. They’ll tell you about stuff but you want to speak to another user and find out what they’re doing: “Hey, how do you find such and such? What do you do there? Isn’t it a nuisance when someone wants to talk to you?” Just swap stories and sometimes you’d just pick up a little pearl of wisdom*.

(Client 18)

Clients also reported hesitations regarding asking speakers to use wireless devices. Client 5, for example, said: “*It’s nerve-racking doing it … I hadn’t been very close to my tutors. So, it’s a weird thing to say: ‘Hey, put this around your neck.’” Clients reported that they “can feel a bit as though you’re invading their [speakers’] space, personal space*” (Client 21). Therefore, clients might prefer a different WMS: “*I’d really like that [WMS], and then I wouldn’t have to ask people to wear the Clip-on Mic … The thing about not having to ask people to wear a microphone. It’s about feeling normal*” (Client 22).

However, client 20 explained that she would persuade the speakers to wear the device and said: “*If it’s a meeting I usually try and con the chairman into putting it around their neck”.* A clinician who is a wireless device user also explained that users should be assertive about their needs.

*I’m not afraid to be assertive. And generally handing the Pen to someone and getting them to speak right into it, in those situations does make it easier. And yeah, you just have to be assertive about your own listening needs*.

(Clinician 9)

Finally, it was clear from the interview data that informal support from family and friends facilitated the use of WMS. For example, the interviewer asked a client whose daughter accompanied her in appointments: “*If your daughter weren’t available to assist you through this process, how would you feel, do you think, in terms of your confidence with getting the device?*”, and the client replied: “*I probably wouldn’t have even been game enough to get it [WMS], I have to admit*” (Client 20).

However, unsupportive SOs could be a barrier to using WMS.

*I still really haven’t done a lot with it [WMS], mainly because I’ve just been, the family I’m with is so uncooperative, uninterested … They don’t even know I’ve got it … I don’t mind using it anywhere except at home. I just really feel put off by the expected reaction I’ll get, so I just don’t, I don’t feel like rocking that boat*.

(Client 13)

## 4. Discussion

This study explored the perceptions and experiences of adults with HI, their SOs, and clinicians regarding the use and benefits of WMS. Data from 43 in-depth qualitative interviews demonstrated that users believe in the benefits of WMS (e.g., better sound quality and improved quality of life). However, there were a number of mediating factors which influenced their successful uptake and use, including the importance of a trial and decision-making process and the need for ongoing training and support to manage issues in different settings. The specific benefits highlighted by the participants in this study support and add to the findings of other studies, with benefits ranging from listening in the distance [17,18] to better sound quality [17] and improvements to hearing in noise [24,25]. The issues identified by the participants in the current study highlight the complexity of managing and adjusting the WMS and were consistent with previous research indicating difficulties with the equipment [17] and issues in some noisy environments [18].

A novel finding of this study was the importance of community understanding of WMS as a facilitator for using the device. Although previous research has reported numerous benefits of WMS [11,17,18], the use of such devices requires the cooperation of communication partners who must also take responsibility for the successful use of the device. The participants in this study reported that members of the public and health professionals often had limited understanding of WMS, meaning they were often reluctant to wear the WMS. In addition to support and understanding from the general community, support from SOs was also identified as an important factor in using WMS. Consistent with previous research highlighting the important influence of SOs [26,27], the participants in the current study described how unsupportive SOs hindered the use of devices.

Another important finding of the current study was the need for more information, counselling, and support for users of WMS. Some of the clients reported not knowing enough about WMS or the variety of situations in which they could use their devices. In addition, SOs were not actively involved in audiology appointments and thus did not receive information about the potential uses and applications of WMS and their important role in the successful use of the device. SOs have to wear the WMS so that the client with an HI can hear them. Therefore, any rehabilitation program must seek to involve SOs more actively throughout the rehabilitation process, including in trial and decision-making appointments. As outlined by previous researchers, more counselling, information provision, and training in line with person- and family-centred principles may increase the use of WMS and other technologies [5,28].

The concept of family-centred care in audiological rehabilitation has been discussed by numerous authors in recent years, with identified benefits to clinicians, clients, and SOs [29,30,31]. There are a range of strategies that can be used to encourage SO attendance and involvement in rehabilitation, with a recent feasibility intervention study demonstrating that when SOs are encouraged to attend appointments by front-of-house staff and when clinicians set up the room for family member involvement and invite families to contribute to the rehabilitation process, there is a subsequent increase in SO attendance and involvement in sessions [31]. These strategies could be applied to encourage greater SO involvement in the introduction and fitting of WMS.

Participants in the current study also reported issues with the complexity of some WMS. Given that HI might be associated with cognitive impairment and accelerated cognitive decline in older adults [32,33], using a complex device may cause frustration and challenges in managing the device. The difficulties in using WMS, as reported by participants in this study, point to the need for more troubleshooting and support services from clinicians and manufacturers. The important role of manufacturers in this ongoing support was highlighted by clients in this study who expressed that they experienced a range of issues in managing their device and that their clinicians were not always able to address these issues. Given that audiologists raised time as a barrier to attending manufacturer workshops, as well as keeping abreast of technological advancements in WMS, a support network for clients by which they can access direct support from manufacturers for troubleshooting may be an effective solution for helping clients troubleshoot technological challenges in a timely manner.

Another finding in this study was the inconsistency in the experiences of different clients regarding background noise and TVs with the same wireless devices. Research indicates that clients experience more hearing difficulties in background noise [1], and WMS are appropriate in the presence of noise [6,8], but some clients in the current study reported that they hear the noise via their WMS, while other clients reported that the WMS cut the noise for them. In using a WMS for TV, again, an inconsistency was reported. Using the WMS blocked surrounding sounds for some clients, but other clients could easily communicate with their SOs while watching TV. The inconsistencies in experiences with using WMS in background noise and for TVs highlighted the importance of providing more information and counseling to clients and more training to clinicians.

Some clients in this study reported that they were reluctant to wear their WMS due to stigma. Both HI and hearing-assistive technologies are associated with stigma [34], and stigma could be associated with any assistive technology use [35]. Stigma is one of the main predictors of the use and uptake of hearing aids [36,37]. However, less stigma might be associated with the use of hearing-assistive technologies for HI; therefore, the management of HI by using devices could have a positive impact [38]. The size and visibility could be the main reasons associated with the stigma of wearing hearing devices, which creates a reluctancy to use them [39]. Therefore, device aesthetics/cosmetics and universal design principles should be considered by manufacturers in producing hearing-assistive technologies [35], as the participants in the current study discussed the visual appeal of WMS.

## 5. Limitations and Future Directions

This research increased our understanding of the experiences of WMS users; however, some limitations of the current study are acknowledged. Some clients reported how unsupportive family members influenced their use of WMS, but none of the unsupportive family members participated in the interviews to help us explore their points of view and the barriers to their support. Community understanding was also identified as an important factor, but we did not recruit people from the community. The broad implementation of WMS can be achieved in communities where public awareness is high. In future research, it would be important to include community members in the interviews as well in order to inform any co-design of health promotion and prevention activities to bring about greater community awareness of HI and WMS. In addition, advocacy groups are encouraged to work with local community leaders to increase awareness about how to communicate with people with HI and increase community understanding of WMS.

Given that using WMS for adults with disabilities other than HI (e.g., dexterity and cognition impairment) was identified as more challenging, it is recommended that future research works develop guidelines and protocols for fitting and follow-up for different groups of clients (e.g., young people, adults with dexterity, and adults with cognitive impairments) in order to make the process of providing WMS more effective and consistent. Further, although clinicians in the current study reported that a successful trial often led to making the decision to use the WMS, varied trial durations (one day to one month) were offered to clients, and a longer trial period was desired by clients. Future research could explore the outcomes of trials to develop evidence-based protocols for trialing WMS for different groups of clients.

## 6. Conclusions

This study explored the experiences of adults with HI, SOs, and clinicians regarding the use of WMS with an in-depth qualitative method. The results of this study have highlighted the complexities of providing and using WMS with adults. However, clients, SOs, and clinicians all reported that, with appropriate experience, expectations, training, and support, WMS can make a real difference in listening and communicating in different situations. The results indicated that there is a real opportunity to introduce WMS to clients and families in a more systematic way, with more ongoing training and support for clients, SOs, and clinicians alike. It is also recommended that SOs be more involved throughout the entire rehabilitation process.

## Figures and Tables

**Table 1:** Demographic Characteristics of Clients (n = 23).

ID	Age (y)	Sex	Education	Employment Status	Living with Person	Years with HI	Years Wearing HA/CI	Type of WMS	Years/Months Using WMS
1	58	female	High school	Full-time	Partner	25	25	Handheld/body-worn WMS and table/group discussion WMS	1 y
2	32	female	Bachelors	Full-time	Partner	32	32	Handheld or body-worn WMS and wireless receivers	7 y
3	54	female	Masters	Part-time	Partner, son/daughter	54	18 (HA), 10 (CI)	Various handheld/body-worn and clip-on WMS	10 y
4	27	female	Postgraduate	Full-time	Alone	27	26	Handheld/body-worn WMS	3 y
5	21	female	High school	Student	Mother	9	7	Handheld/body-worn WMS	2 m
7	65	male	Bachelors	Retired	Partner	65	15	Handheld/body-worn WMS and wireless receivers	3 m
8	66	male	TAFE/trade	Retired	Partner	30	18 (HA), 1 (CI)	Handheld/body-worn WMS and wireless receivers	8 m
9	71	male	Bachelors	Retired	Alone	57	57 (HA), 2.5 (CI)	Cochlear accessories—Trialled friend’s handheld/body-worn WMS	*
10	60	female	Postgraduate	Casual	Other relatives	60	19	Handheld/body-worn WMS	3.5 y
11	80	male	*	*	Partner	73	70	Handheld/body-worn WMS	*
12	80	female	Postgraduate	Retired	Partner	Since mid-late 80s	Since mid-late 80s	Handheld/body-worn WMS	*
13	77	female	University certificate	Retired	Son/daughter	57	11 (HA), 2 (CI)	Handheld/body-worn WMS and wireless receivers	1.5 y
14	49	female	Postgraduate	Part-time	Partner, son/daughter	37	37 (HA), 6 (CI)	Handheld/body-worn WMS	3.6 y
15	64	female	Bachelors	Part-time	Alone	64	20	Handheld/body-worn WMS	5 y
16	30	male	Bachelors	Full-time	Partner	30	30 (HA), 5 (CI)	Handheld/body-worn WMS and wireless receivers	2 y
18	57	male	Diploma	Part-time	Partner	30	5	Handheld/body-worn WMS	3 y
19	46	male	Bachelors	Full-time	Partner, son/daughter	33	27 (HA), 2 (CI)	Handheld/body-worn WMS and wireless receivers	4 y
20	*	female	*	*	*	*	*	Handheld/body-worn WMS	*
21	71	female	Postgraduate	Retired	Partner	9	7	Various handheld/body-worn WMS and wireless receivers	6 y
22	56	male	Diploma	Full-time	Partner, son/daughter	*	*	Handheld/body-worn WMS and wireless receivers	7 y
23	37	female	Bachelors	Full-time	Partner, son/daughter	*	8 (CI)	Handheld/body-worn WMS	4 y
24	67	female	Masters	Retired	Housemate	67	20	Handheld/body-worn WMS	5 y
25	52	male	Masters	Freelance	Partner	6	6	Various handheld/body-worn WMS	*

Note: HL: hearing loss, HA: hearing aids, CI: cochlear implant, WMS: wireless microphone system, y: years, m: months, *: missing data. Roger Pen and EasyPen = handheld/body-worn wireless microphone. Roger Table mic = wireless microphone for group discussions. Roger X and MyLink = wireless receiver for HA and CI.

**Table 2:** Theme 1. With experience and clear expectations, users believe WMS can make a difference.

Categories	Subcategories
Clients, significant others, and clinicians believe in the benefits of WMS	
Clients “looking to bridge the gap” and make a difference in their lives	
WMS can be used in many situations	Meetings and work environment
	Education settings
	Noisy situations
	Phones, mobiles, and iPads
	TV, radio, computers, and laptops
	Healthcare services and nursing homes
	Hearing over distance
	Inside home
	Social activities
	Sport and physical activity
	Transport
	When lip reading is not possible
	Clinicians like the versatility and multi-functionality of the WMS
Clinicians had varied experiences with WMS	Clinicians’ experience and skills
	Clinicians believe some groups of clients are more successful users of WMS
Expectations matter	Clients’ and significant others’ high expectations
	Clinicians try to form realistic expectations for clients
	WMS meet the expectations of some users
Community understanding	Weak public awareness about assistive technologies and communication with people with hearing loss
	Health professionals may not know how to interact with people with HI and refuse to use WMS
	Teachers’ and universities’ awareness about WMS
	Phonak awareness

**Table 3:** Theme 2. The trial and decision-making process is important.

Categories	Subcategories
When and how WMS systems get introduced	Clinicians identify potential users based on clients’ needs and goals and the lack of success with amplification alone
	Heard about wireless microphone systems from clinicians or other resources
	Marketing materials
	Impact of online reviews on decision making
Clients’ goals and needs inform the trial and decision making	
Trialling WMS informs expectations and illustrates what it might do	Trial sells the device
	Opportunities for a trial to illustrate what wireless microphone systems might do
	Trialling helps clients to form their expectations
	Trialling in different situations is important
	Clinicians offer varied trial durations
Cosmetic considerations influence clients’ decision making	
Compatibility of WMS with other devices increases the interest in it	
Significant others are variably involved in the trial and decision-making process	Clinicians’ desire to involve significant others
	Significant others may not receive information about WMS and may not be involved in decision making
“Cost is quite prohibitive for some people”, while the availability of funding motivates the clients	

**Table 4:** Theme 3. Clients’ experiences using WMS.

Categories	Subcategories
Better sound quality than what comes through the hearing aids	
Hearing a lot more with less listening fatigue	
Feeling more confident, independent, included, and engaged when using WMS	
Improves the quality of life and lifestyle	
Improvement in the families’ situations	
Clients use both captioning and WMS	
Learning by trial and error	
Clients face a variety of people’s reactions to WMS	Acceptant and supportive reactions
	Unsupportive reactions
	Clients use different approaches to get speakers’ cooperation in order to facilitate using WMS
Not all clients use WMS consistently	

**Table 5:** Theme 4. Issues with WMS and technology.

Categories	Subcategories
Ongoing challenges with communication situations	WMS are better in one-on-one situations than in multiple interactions
	Experiencing difficulties in meetings and lectures
	Hearing the noise rather than the speaker
	WMS blocks surrounding voices and locks to the speaker
Design and technical issues	Small buttons
	Understanding the message through the LEDs is challenging
	Battery charge is challenging
	Wireless devices keep dropping out
	Unnatural sound quality
	Concerns about security and durability
	Health-related concerns
WMS are complex for users	WMS can be complicated and difficult to manage
	Challenges with phone
	Using WMS with TVs is complex, and working out how to set it up to the TV is a “nightmare”
	Hassle of having an extra device
	Frustrated from using WMS
Users compare different devices and services	

**Table 6:** Theme 5. Users require ongoing training and support to use wireless microphone systems.

Categories	Subcategories
Users require more information	Clients not aware of the benefits of WMS and the different situations in which wireless devices could be used
	Clients require more counselling and information
	More awareness of significant others regarding HI and hearing technologies is required
	Additional instructions are required
Clinicians require support	A lot of counselling and tweaking is required for fitting WMS
	A variety of TVs and phones make the fitting challenging for clinicians
	Clinicians require refreshers and training about WMS
Ongoing support for clients	Clinicians help with setting up the TV, phone, and meeting rooms
	Lack of a good follow-up for some clients
	Learning and remembering how to use the device
	“There needs to be a longer warranty period in line with a lot of other companies”
	A desire for a direct line for troubleshooting and access to a contact person for practical support, as the current troubleshooting is not desirable for all clients
	A desire for connecting with other WMS users
	Hesitation to ask speakers to use WMS
Significant others influence outcomes	Informal support from family and friends facilitates using WMS
	Unsupportive significant others are a big barrier to using WMS

## Data Availability

Not applicable.

## Appendix A

**CLIENT INTERVIEW GUIDE**

*I. USER EXPERIENCE*

(a) **Can you tell me about your experience of using wireless microphone systems?**
(b) **How do you normally use your device?**
  ○ What situations do you use your device in?
  ○ How often?
  ○ Why?

*II. BENEFITS & CHALLENGES*

(c) **What does the device do for you?**
  ○ What are the major advantages?
  ○ How is it different from using your HA/CI alone?
(d) **How do you find using the device in different situations or with different people?**
  ○ Is there anything that is particularly helpful or unhelpful?
  ○ Is there anything that is particularly challenging or stressful?
  ○ Is there anything you don’t like about using the device?
  ○ Are there any situations where you would avoid using your device?
(e) **Does the device meet your expectations and needs?**
  ○ Does it help you in the way you had hoped?
  ○ If you could change something about it, what would it be?
(f) **Why did you stop using your device?** (If applicable)
(g) **Have you family/friends/colleagues made any comments about the device?**

*III. REASONS & MOTIVATIONS*

(h) **How did you first find out about the device?**
  ○ How did you first talk about it with your audiologist?
  ○ How did your audiologist first introduce the device to you?
(i) **What were your reasons for getting the device?**
  ○ What were the things you considered or talked about when deciding?
  ○ What were the most important things that led you to get the device?
  ○ Was there anyone or anything that had a big influence on your decision?
  ○ Was there any family member/partner involved in this decision?
(j) **How was the process of getting the device?**
  ○ What were the steps involved in getting the device?
  ○ What were the steps involved in setting it up and figuring out how to use it?
  ○ How did you decide on this particular device?

*IV. INFORMATION, TRAINING, SUPPORT*

(k) **How do you feel about the support you’ve received for using the device?**
  ○ How supported were you at the beginning when you first got the device?
  ○ What has been most helpful or unhelpful in terms of support and information?
  ○ Where has the support and information come from?
  ○ Is there anywhere else you go to for support and information?
  ○ Did your family members/communication partners receive any support?
(l) **Was there anything missing from the support or information available to you?**
  ○ Are there any situations when you need more help?
  ○ Are there any difficulties in getting help when you need it?
  ○ Did you come up with any of your own strategies to troubleshoot?
  ○ Is there any ongoing support or information?

**SIGNIFICANT OTHER INTERVIEW GUIDE**

*I. USER EXPERIENCE*

(a) **What’s your experience with communicating with someone who has a hearing impairment?**
  ○ Who do you communicate with?
  ○ How often and in what situations?
  ○ What is their level of hearing difficulty?
(b) **Can you tell me about your experience of using a wireless microphone system?**
(c) **How do you normally use the device?**
  ○ What situations do you use the device in?
  ○ How often?
  ○ Why?

*II. BENEFITS & CHALLENGES*

(d) **What does the device do for you?**
  ○ What are the major advantages?
  ○ How is it different when you don’t use it?
(e) **How do you find using the device in different situations?**
  ○ Can you describe a time when it worked really well?
  ○ Can you describe a time when it didn’t work so well?
  ○ Is there anything that is particularly helpful or unhelpful?
  ○ Is there anything that is particularly challenging or stressful?
  ○ Is there anything you don’t like about using the device?
  ○ Are there any situations where you would avoid using the device?
(f) **Does the device meet your expectations or needs?**
  ○ Does it help in the way you had hoped?
  ○ If you could change something about it, what would it be?
(g) **Why did you stop using the device? (If applicable)**

*III. REASONS & MOTIVATIONS*

(h) **How did you first find out about the device?**
  ○ Had you known anything about wireless microphone systems before?
  ○ Had you had any experience with using wireless microphone systems before?
(i) **How involved were you in the decision to get the device? (If applicable)**
  ○ Why did you decide to get the device?
  ○ What did you hope it would do for you?
  ○ What were the main things you considered and talked about beforehand?
  ○ Is there anything else you would have wanted to know at the time?

*IV. INFORMATION, TRAINING, SUPPORT*

(j) **How do you feel about the support you’ve received for using the device?**
  ○ How supported were you at the beginning when you first got the device?
  ○ What has been most helpful or unhelpful in terms of support and information?
  ○ Where has the support and information come from?
  ○ Is there anywhere else you go to for support and information?
(k) **Was there anything missing from the support or information available to you?**
  ○ Are there any situations when you need more help?
  ○ Are there any difficulties in getting help when you need it?
  ○ Did you come up with any of your own strategies to troubleshoot?
  ○ Is there any ongoing support or information?

**CLINICIAN INTERVIEW GUIDE**

*I. KNOWLEDGE AND EXPERIENCE*

(a) **Can you tell me about your experience with wireless microphone systems with adults?**
  - How long have you been working with the devices with adults?
  - Which types of devices have you worked with?
  - When and where was your first exposure to using the devices with adults?

*II. RECOMMENDING, FITTING, SUPPORT*

(b) **What’s your process for recommending a device to a client?**
  ○ How do you identify suitable clients?
  ○ What kind of client do you think benefits most?
  ○ At what stage and how would you introduce a device?
  ○ Where do you see the devices sitting in the range of hearing support solutions?
(c) **What’s your process for fitting a device with a client?**
  ○ What are the steps?
  ○ Who else do you involve?
  ○ How do you support their decision-making?
  ○ What information do you provide?
  ○ What practical support do you provide
(d) **How do you support clients to use their device?**
  ○ What approaches tend to work best?
  ○ What does your management and support entail after the initial fitting process?
  ○ What are the main challenges?

*III. BENEFITS, BARRIERS, FACILITATORS*

(e) **How do you feel about using wireless microphone systems with adults?**
  ○ How effective do you think the devices are in meeting client needs?
  ○ What are the main benefits for clients and their communication partners?
  ○ Do the devices meet your expectations?
  ○ Can you describe a time when the device has worked really well?
(f) **What holds you back from recommending a device?**
  - Can you describe a time when the device hasn’t worked so well?
  - If you could change something about the devices, what would you change?
(g) **What are the differences between clients who use the devices effectively and those who don’t?**
  - Why do you think some clients stop using their device?

*IV. PROFESSIONAL TRAINING & SUPPORT*

(h) **What training and support have you received for fitting and managing these devices with adults?**
  - Who or where can you go to for additional support or training?
  - What professional development is available?
  - What avenues do you typically access?
(i) **How could Phonak best support you in using these devices with adults?**
  - What do you find helpful or unhelpful?
  - Are there differences in the support you receive from different manufacturers?
  - In future wireless microphone systems training and information, what would you like the manufacturer to cover?
